# Contributions of lifestyle, education, and cardiovascular risk factors to the brain age gap

**DOI:** 10.1016/j.nbas.2025.100149

**Published:** 2025-08-11

**Authors:** Kostas Stoitsas, Pieter Bakx, Trudy Voortman, Jing Yu, Gennady Roshchupkin, Daniel Bos

**Affiliations:** a Erasmus Medical Center Department of Epidemiology, Rotterdam, The Netherlands; b Erasmus University Rotterdam Erasmus School of Health Policy and Management, Rotterdam, The Netherlands; c Erasmus Medical Center Department of Radiology and Nuclear Medicine, Rotterdam, The Netherlands

**Keywords:** Brain age gap, Longitudinal study, Determinants of brain age

## Abstract

The brain age gap is the difference between chronological age and the age predicted from Magnetic Resonance Imaging (MRI) brain scans. We investigated the influence of life habits and cardio-metabolic factors on this gap. A convolutional neural network (CNN) was trained on structural MRI scans from dementia-free participants in the Rotterdam Study.

Scans were collected every 3–4 years from 2005 to 2016. 10,989 images from 5,167 participants (mean age: 64 years [range: 45–98], 54 % female) were used to train and evaluate the model. We run analysis of variance and linear mixed models to assess associations between brain age gap and smoking, sleep, alcohol consumption, education, and cardio-metabolic factors.

The brain age gap in participants who developed dementia was elevated relative to cognitively healthy individuals and showed a progressive increase throughout the study period.

We found that together, the examined factors explained no more than 21% of the variance in brain age gap. Smoking, alcohol consumption, and elevated glucose levels are significantly associated with an increased brain age gap, consistent with earlier studies linking these factors to brain atrophy and cognitive decline.

## Highlights

• Major lifestyle and cardio-metabolic factors account for no more than 21% of the variance of the brain age gap.
• Smoking, alcohol, age, and glucose levels were most prominently associated with the brain age gap.
• Education, sleeping, cholesterol and blood pressure were not associated with brain age gap.

## Graphical abstract

**Figure:**
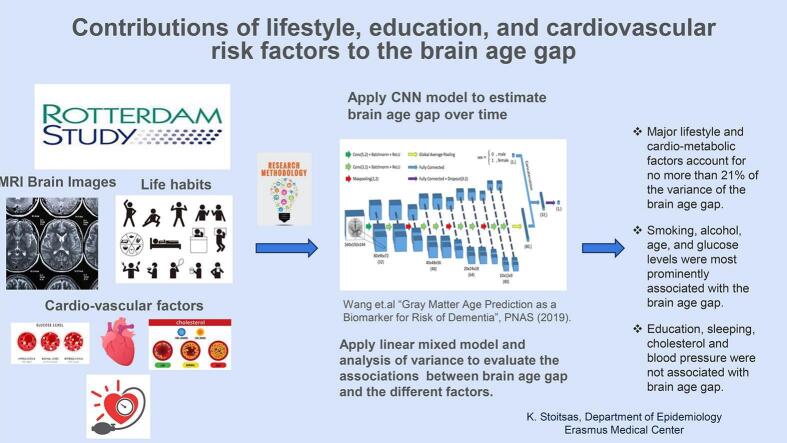

## 1 Introduction

Brain age gap, also known as delta brain age, is defined as the difference between an individual’s chronological age and their estimated brain age as derived from neuroimaging data. The brain age gap is an estimate of how an individual’s brain is aging relative to their peers [1]. The brain age gap has shown promise in predicting long-term outcomes such as dementia, bipolar disorder, schizophrenia and cognitive decline, making it a valuable tool for both research and clinical applications [2].

Genetic influences, environmental factors and lifestyle habits such as diet, exercise, and sleep quality play a crucial role in brain aging [3]. Regular physical activity and a heart-healthy diet have been linked to slower brain aging and a reduced risk of neurodegenerative diseases [4]. Cardiovascular health is particularly important, as conditions like hypertension and diabetes can accelerate brain aging by affecting blood flow [5]. Although previous studies have examined the association between these factors and brain aging, only a few examined associations with the brain age gap [6]. The data in these studies was limited to small and homogeneous study samples, lack of longitudinal data, and limited consideration of modifiable factors such as lifestyle and cardiovascular health [7].

Given the importance of brain age gap as a predictor of cognitive health and neurodegenerative diseases, we explored how this parameter is associated with known modifiable factors for dementia risk, such as cardiovascular health, lifestyle habits, and education. Lifestyle and cardio-metabolic data were collected alongside neuroimaging data, allowing us to investigate associations between these variables and brain age gap longitudinally. Analysis of variance and linear mixed models were used to quantify the influence of these factors. Our findings contribute to the growing body of evidence that targeting modifiable risk factors may provide strategies to maintain lower brain age gap and promote healthier cognitive aging [8].

## 2 Methods

### 2.1 General methodology

In this study, we estimated the brain age gap longitudinally over a 12-year period. To do this, we trained a deep learning model using MRI brain images from healthy individuals who did not develop dementia before or during the study. Only participants with at least one MRI scan during the study period were included in the training set, and all available MRI scans for these individuals were used. Once the model was trained and evaluated, we applied it to estimate the brain age gap across time for both healthy individuals and those diagnosed with dementia. This analysis used MRI images excluded from the training set, including those from validation, test datasets, and a subset of dementia cases, to minimize model bias. Alongside brain age gap measurements, we collected longitudinal data on lifestyle, education, and cardiovascular factors. We then conducted analysis of variance and regression analyses (applying time varying models such as linear mixed models) to determine the proportion of variance in brain age gap explained by these factors, as well as to identify which ones had significant effects (See Fig. A9).

### 2.2 Sample

To select the parameters which have an impact on the brain age gap, we relied on previous literature demonstrating their association with brain aging and cognitive decline. For instance, studies have shown that excessive alcohol intake and smoking are linked to accelerated brain aging and structural brain changes [9]. Cardiovascular risk factors, including elevated blood glucose and hypertension, have also been robustly connected to increased brain age [10], [11]. These established findings guided the selection of variables in our analysis.

We use the Rotterdam Study to get longitudinal data related with life habits, education, cardio-metabolic factors and MRI brain images over time. Rotterdam Study is a prospective cohort study conducted in the Ommoord district of Rotterdam, the Netherlands since 1990. The primary objectives of the Rotterdam Study are exploring risk factors associated with cardio-vascular, neurological, ophthalmological, and endocrine conditions in the elderly population. In 2008, the study had enrolled approximately 15,000 individuals 45 years of age or older. Participants underwent extensive interviews and examinations, including sample collections for detailed molecular and genetic analyses. These examinations were periodically repeated every 3–4 years to account for potential changes in participants’ characteristics [12].

We take 1 January 2005 as the baseline for our study because MRI brain images were collected between 16 August 2005 and 19 April 2016. We included participants of the Rotterdam Study who had at least one MRI image (Fig. A.9). 129 participants were not eligible for the study due to artifacts or cortical artifacts. Data on life habits and cardio-vascular factors were missing for 14.2 % of the sample and were imputed using the “MICE” package in R [13] (see Table A.2 for a comparison between unimputed and imputed data).

The dataset was initially split into four sets. First, subjects with incident dementia (403 subjects and 638 images, Mean Age: 76.82, Female: 57.05 %) were placed in a separate dataset. Subsequently, the subjects with no incident or prevalent dementia were split in three sets: training (4667 subjects with 9631 images, Mean Age: 64.03, Female: 54.28 %), validation (300 subjects with 803 images, Mean Age: 64.76, Female: 54.55 %), and test (200 subjects with 555 images, Mean Age: 64.55, Female: 53.67 %). The validation and test sets included participants for whom data at least two time points over the study period, allowing for the effective application of linear mixed models.

To improve the model predicting accurately at extreme ages (below 47 and above 85), 5000 images were sampled with replacement. For subjects aged between 47 and 85 years old, we down-sampled 5000 observations without replacement. In total, the final training dataset contains 10,000 observations. Due to down sampling, 2105 participants with 3901 images were excluded from the training set. These excluded participants together with those from validation, test and participants with incident dementia were placed in a 5th data set for analysis with linear mixed models (3008 subjects with 5897 images, Mean Age: 67.22, Female: 53.80 %).

### 2.3 MRI brain images

Multi-parametric MRI brain data were acquired using a 1.5 T GE Signa Excite MRI scanner [14]. FreeSurfer version 6.0 [15] was used to segment supratentorial gray matter (GM) based on the T1-weighted brain MRI images [16]. GM density maps were computed based on an optimized voxel-based morphometry (VBM) protocol using the FSLVBM pipeline [17], [18]. First, all GM maps were non-linearly registered to the standard Montreal Neurological Institute GM probability template (ICBM 152 Nonlinear atlases version 2009) with a 1 × 1 × 1 mm voxel resolution. Second, a spatial modulation procedure was used to compensate for differences in absolute GM volume due to the registration. This is achieved by multiplying voxel density values by the Jacobian determinants estimated during registration. The matrix size of the modulated GM density maps was 196 x 232 x 188. We did not apply smoothing on the VBM results because smoothing is a subgroup of possible mathematical operations which the network filters in the convolutional layer can represent. We performed a quality control based on the proportion (5 %) of outlier voxels and an additional manual check to exclude the outliers. Finally, we applied cropping and padding on the images to cut proper the edges, and masked the images with a k-Nearest Neighbor-classifier segmented GM mask [19]. The matrix size of the final images was 160 × 192 × 144. Structural MRI brain images were recorded every 3–4 years. There are 784 participants with 1 observation, 1620 subjects with 2 observations, 543 subjects with 3 observations and 61 participants with 4 observations.

### 2.4 Life habits and education

We used information on alcohol consumption (grams per day), sleeping (sleeping hours per night) and smoking (binary indicator for smoking cigar, pipe or cigarette). Educational attainment was classified using the international standard classification of education [20]. We aggregated the top four of the seven levels into a single category, resulting in four levels: (1) primary education, (2) low level vocational training (3) medium level secondary education (4) medium level vocational training to university level [21]. The educational categories were aggregated to reflect meaningful educational strata within the Dutch education system while maintaining sufficient group sizes for robust statistical analysis — not because they were all considered high-level education.

### 2.5 Cardio-metabolic factors

We included the following cardio-metabolic risk factors: Diastolic blood pressure (mm Hg), Systolic blood pressure (mm Hg), Glucose (mmol/l), Cholesterol (mmol/l). Furthermore, we include indicators for using anti-cholesterol medication, anti-hypertensives and anti-diabetic medication.

### 2.6 Calculation of brain age gap and Statistical analysis

To calculate the brain age gap, we build on the approach of [2]. We implemented a convolutional neural network (CNN) with four to six convolutional blocks. Input to the 3-dimensional (3D) regression CNN model is 3D grey matter density maps from VBM. Next to the 3D grey matter density map, the sex of the participant is provided to the model. This provides to the model the ability to adjust for GM differences between male and female subjects. The original model consists of four convolutional blocks. Experiments were performed by adding one and two extra convolutional blocks. The learning rate was initially at 0.001 and was reduced during experimentation to 0.0005. Additionally, the number of neurons at the output layer was varied between 32 and 256.

Prediction accuracy was assessed based on the results in test set. Model accuracy was measured based on the mean absolute error (MAE) of prediction, i.e., the difference between model output and real chronological age (gap = age brain predicted–age chronological). The best model was selected based on its performance on the test set. The training of the model is executed on the minimization of the root mean squared error (RMSE) of the gap, as RMSE penalizes outliers more than MAE.

The model was over predicting at lower ages and under predicting at high ages for healthy participants. To overcome this issue, two actions were taken: we oversampled and down sampled observations, as described under Sample section. Furthermore, we applied the correction to the test set proposed by [22], [23]. According to this methodology, the error observed at the validation dataset generalizes to the test data set. Thus, we fit a straight line between the predicted brain age and the chronological age in the validation set, and then apply the fitted slope and intercept to correct biased predictions in the test set (corrected values = (model values − intercept)/slope. To evaluate the bias correction, Spearman correlation of the residuals versus chronological age of the participants was calculated. Python and the package of Keras was applied to train and evaluate the model [24].

We measured the partial variance explained by each predictor by taking the difference between the *R*^2^ of a model including all predictors and the *R*^2^ of a model where the predictor is removed [25].

We evaluated the association between brain age gap and various factors using linear mixed models on the full study sample (3008 participants with 5897 images). We estimated two linear mixed models: one with life habits and education as independent variables and one with cardio-metabolic factors as independent variables because of the nature and impact of these variables on the prediction of health outcomes. Lifestyle habits and education are significant social and behavioral factors that influence long-term health. In contrast, cardio-metabolic factors have a more direct impact on overall health. The linear mixed models included random effects at the participant level (random intercepts) to account for repeated measures within individuals [26]. Furthermore, both models included i) time from inclusion in the study and ii) age at the scan date as independent variables.

## 3 Results

### 3.1 Sample characteristics

The full sample contains N = 5570 individuals of mean age of 66.46 years and percentage female 54.35 %. The subsample used for the linear mixed models (regression sample) contains N = 3008 individuals with mean age of 67.22 years and percentage female 53.80 %. Descriptive statistics of the two samples are presented in Table 1. Statistical tests show that both sets contain participants with similar characteristics (*Statistical Tests, A1*), except for smoking status.

**Table 1:** Descriptive statistics: full sample and subsample used in the regression analysis.

**Characteristic**	**Full sample**	**Regression sample**
Age (mean, sd)	(66.46, 10.03)	(67.22, 8.82)
Sex Percentage Female	54.35 %	53.80 %
Alcohol gr per day (mean, sd)	(9.35, 11.38)	(9.68, 11.58)
Sleeping hrs per night (mean, sd)	(6.90, 1.25)	(6.88, 1.25)
Smoking percentage	30.67 %	33.23 %
High education percentage	22.91 %	22.06 %
Systolic blood pressure in mm Hg (mean, sd)	(139.61, 20.21)	(136.96, 19.72)
Diastolic blood pressure in mm Hg (mean, sd)	(81.37, 11.31)	(81.76, 11.06)
Glucose mmol/lt (mean, sd)	(5.63, 1.34)	(5.72, 1.19)
Cholesterol mmol/lt (mean, sd)	(5.62, 1.05)	(5.67, 1.02)
Observations	11,627	5897
Unique individuals	5570	3008

### 3.2 Optimization of the deep neural network

We achieved the lowest MAE (3.75) for the validation set with a low learning rate (0.0005), two extra blocks (in total six convolutional blocks) and 256 neurons at the output layer. During the optimization, the Spearman correlation of the residuals of the validation and test set improved from −0.67 to −0.42.

After the correction for the bias at low and high ages, the MAE for the test set increased from 3.88 to 4.12 while the Spearman correlation for residuals versus the chronological age decreased from −0.47 to −0.02.

### 3.3 Explained variance

Table 2 shows that chronological age explained most of the variance of brain age gap with 12.52 %. Smoking, alcohol consumption, glucose levels, and blood pressure also contributed to explaining the variance. Collectively, all factors contributed to 21 % of the total variance.

**Table 2:** Brain age gap variance explained by lifestyle factors, age, education, sex and cardiovascular factors.

**Age, life habits, education and sex**	**(R**2**%)**	**Cardiovascular factors**	**(R**2**%)**
Chronological age	12.52	Systolic blood pressure	0.95
Smoking	1.15	Diastolic blood pressure	0.84
Alcohol gr per day	1.36	Anti-hypertensives medication	0.22
Sleeping hrs per night	0.29	Glucose level in blood	1.70
Education	0.23	Anti-diabetic medication	0.40
Sex	0.25	Cholesterol	0.40
		Anti-cholesterol medication	0.10

Note: A model with all the variables explains 20.41 % of the variance of brain age gap. N = 5897 (5259 for 2605 unique healthy participants; 638 for 403 unique participants with dementia).

### 3.4 Association of the brain age gap with life habits, education and cardiometabolic factors

Increased brain age was associated with higher chronological age (β = 0.13, p < 0.05), smoking (β = 0.40, p < 0.05) and alcohol consumption (β = 0.01, p < 0.05) (Table 3). Participants who smoke had a 0.5-year larger brain age gap than non-smokers. Besides, alcohol increased brain age gap by 0.01 years by each gram of consumption.

**Table 3:** Linear mixed model results for lifestyle factors, education and sex.

	**Estimate**	**Std. Error**	**95 % CI**	**t value**	**p value**
Intercept	−8.651	0.763	[-10.14, −7.15]	−11.331	*<* 0*.*001
Time since inclusion	0.012	0.025	[-0.037, 0.061]	0.687	0.492
Age at scan	0.132	0.013	[0.106, 0.158]	12.884	*<* 0*.*001
Smoking	0.408	0.135	[0.143, 0.673]	3.092	0.002
Alcohol per day	0.012	0.005	[0.002, 0.022]	2.265	0.027
Sleeping hrs per night	0.038	0.043	[-0.046, 0.122]	0.804	0.413
Education	0.021	0.085	[-0.145, 0.187]	0.332	0.737
Sex	0.147	0.202	[-0.249, 0.543]	0.684	0.492

Note: N = 5897 (5259 for 2605 unique healthy participants; 638 for 403 unique participants with dementia).

A higher brain age gap was furthermore associated with higher glucose levels (*β* = 0.15, *p <* 0*.*05) and use of anti-diabetic medication (*β* = 1.31, *p <* 0*.*05) (Table 4). Moreover, brain age gap was positively associated with chronological age (*β* = 0.13, *p <* 0*.*001).

**Table 4:** Linear mixed model results for cardio-metabolic factors.

**Effect**	**Estimate**	**Std. Error**	**95 % CI**	**t value**	**p value**
Intercept	−9.343	0.921	[-11.14, −7.53]	−10.125	*<* 0*.*001
Time since inclusion	0.002	0.018	[-0.033, 0.037]	0.122	0.907
Chronological age	0.135	0.014	[0.108, 0.162]	11.856	*<* 0*.*001
Systolic blood pressure	0.005	0.004	[-0.003, 0.013]	1.112	0.266
Diastolic blood pressure	0.001	0.008	[-0.015, 0.017]	0.122	0.907
Anti-hypertensive medication	−0.343	0.866	[-2.040, 1.354]	−0.408	0.699
Glucose level in blood	0.154	0.055	[0.046, 0.262]	2.586	0.009
Anti-diabetic medication	1.313	0.335	[0.656, 1.970]	3.963	*<* 0*.*001
Cholesterol	−0.021	0.077	[-0.172, 0.130]	−0.424	0.665
Anti-cholesterol medication	0.043	0.164	[-0.278, 0.364]	0.283	0.776
Sex	0.113	0.204	[-0.287, 0.513]	0.585	0.554

Note: N = 5897 (5259 for 2605 unique healthy participants; 638 for 403 unique participants with dementia).

## 4 Discussion

The brain age gap, the difference between chronological age and brain-predicted age from MRI scans, is a biomarker for neurodegeneration. This study investigated the association between the brain age gap and lifestyle, education and cardio-metabolic factors. The brain age gap was calculated using a CNN trained on MRI brain images from healthy participants. Although brain age gap has gained popularity as a biomarker of brain health, recent evidence suggests that it may not robustly capture longitudinal changes in brain structure [27]. This raises important questions about the interpretability and utility of brain age gap as a dynamic marker. Specifically, it remains unclear whether changes in modifiable risk factors—such as smoking, alcohol consumption, or blood pressure control—can actually lead to measurable improvements in brain age or merely reflect baseline differences. The present study investigates whether lifestyle modifications produce corresponding shifts in brain age estimates over time, which would support its use as a responsive biomarker for intervention outcomes.

Based on our analysis we report four main findings. First, [27] rigorously tested whether cross-sectional brain age gap predicts its longitudinal change and found no meaningful associations. They argue that brain age gap predominantly reflects stable, early-life brain characteristics (e.g., birth weight, genetic makeup). If longitudinal brain age gap change does not correlate with cross-sectional brain age gap, this may imply that: (1) Cross-sectional brain age gap reflects a baseline deviation from normative aging rather than an individual’s brain aging trajectory. (2) Individuals with a high or low brain age gap at one time point do not necessarily exhibit accelerated or decelerated brain aging over time. (3) Different mechanisms—such as genetics, lifestyle, disease processes may govern baseline brain age gap and its longitudinal dynamics.

**Fig. A.4:**
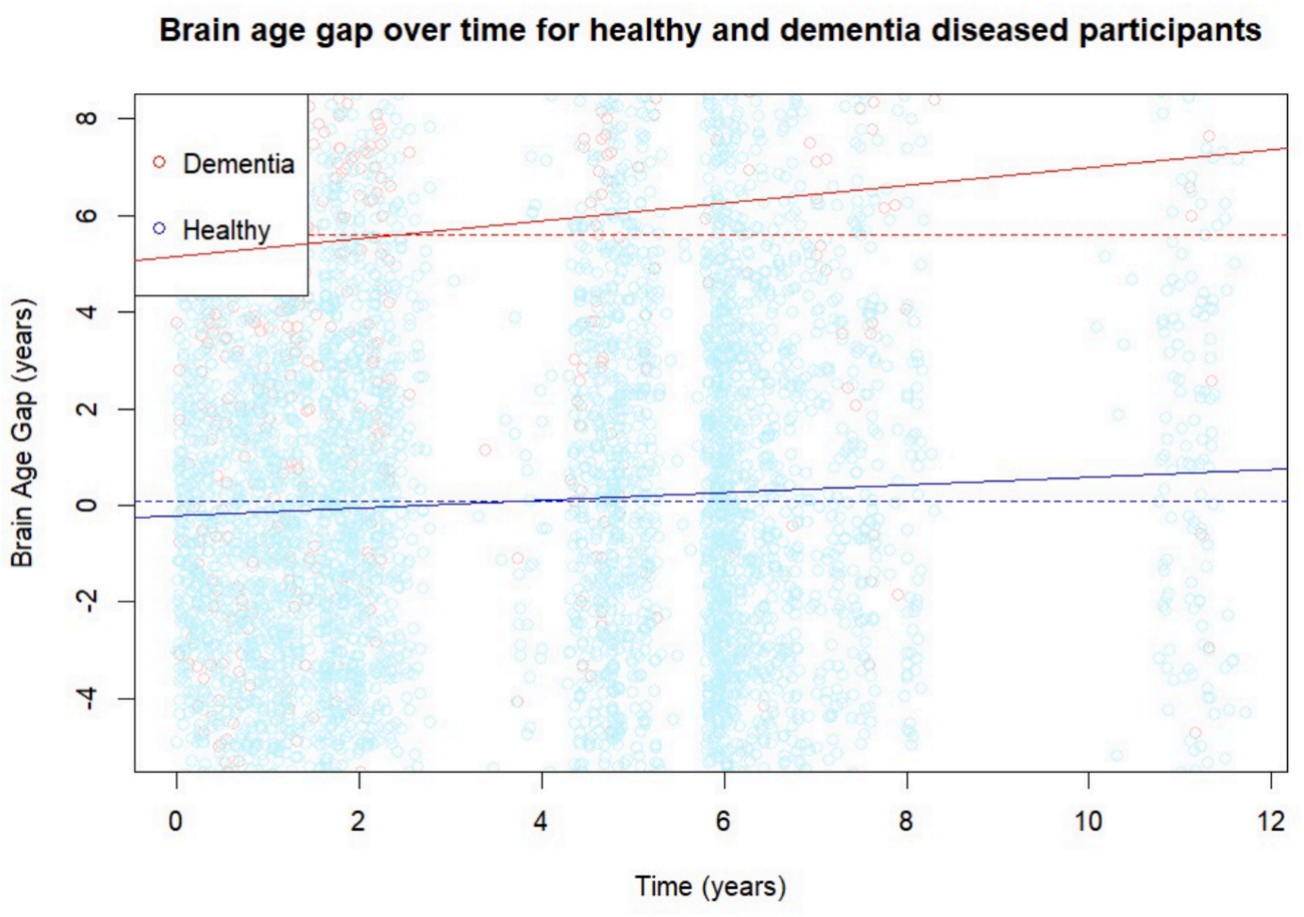
. By fitting the data with linear regression we observe that for healthy participants the brain age gap is stable and close to zero (blue solid line). On the contrary, for participants who developed dementia the average value of brain age gap (dashed red line) is larger than the average value of healthy subjects (dashed blue line). Additionally, the brain age gap for people who develop dementia increases over time (red solid line). A Chow test returns an F value of 2.01 with a p-value of 0.002 indicative that the regression coefficients (intercepts and slopes) are significantly different between healthy and subjects who develop dementia during the study. This means the relationship between Age Gap and time of the study varies across the two datasets (healthy and dementia). 2605 Healthy Participants with 5259 images and 403 Dementia Participants with 638 images

**Fig. A.5:**
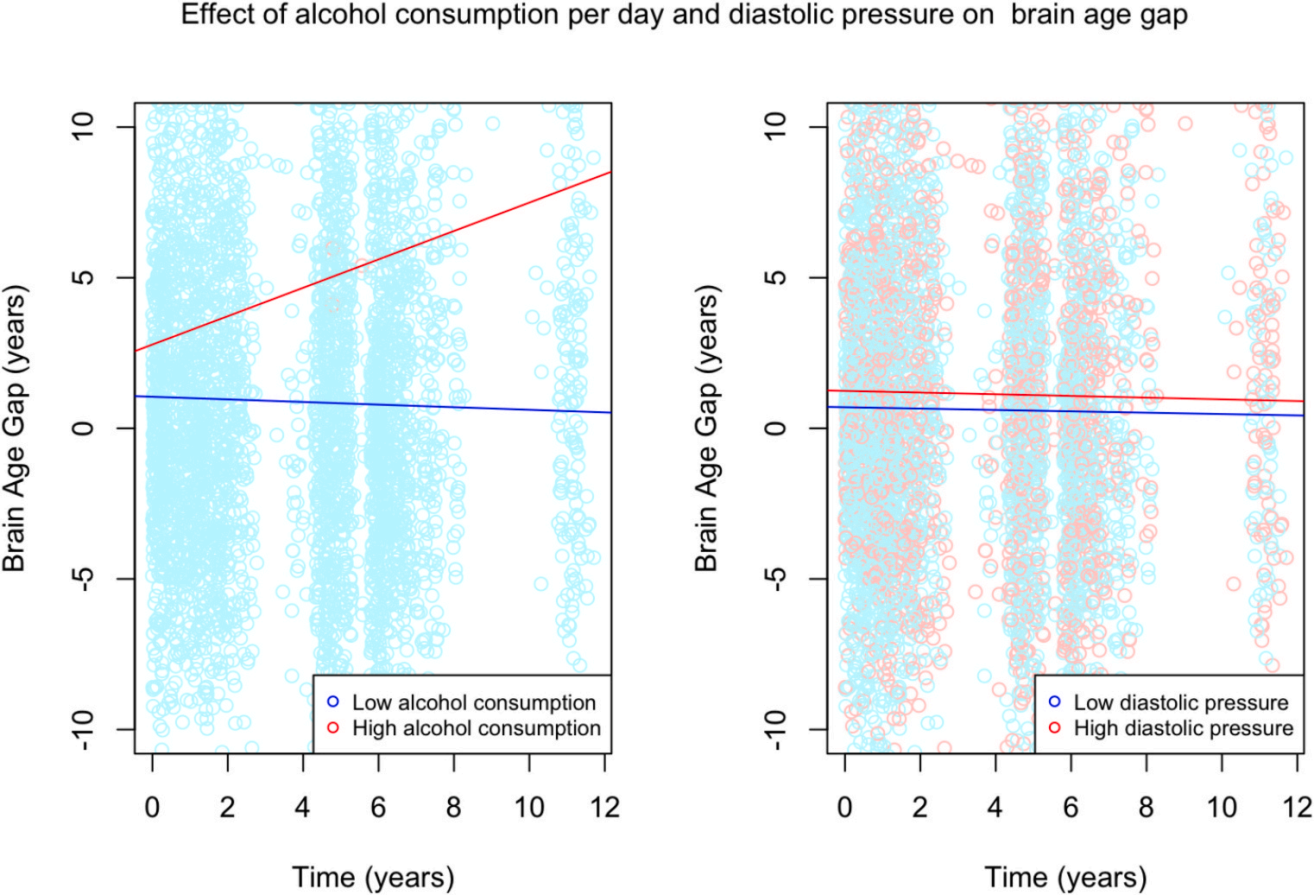
. Effect of alcohol on brain age gap (left). 100 gr per day of alcohol is considered as the threshold for high consumption. Effect of diastolic pressure on the right (as threshold for high pressure is considered the value of 85) The assumptions of the multivariate linear regression for homoscedasticity and normality of the residuals is assessed by plotting the residuals over the fitted values and the density plot Figure Appendix A.6 and Figure Appendix A.7. Residuals are fitted with a straight line close to zero while the density plot is close to a bell shaped.

**Fig. A.9:**
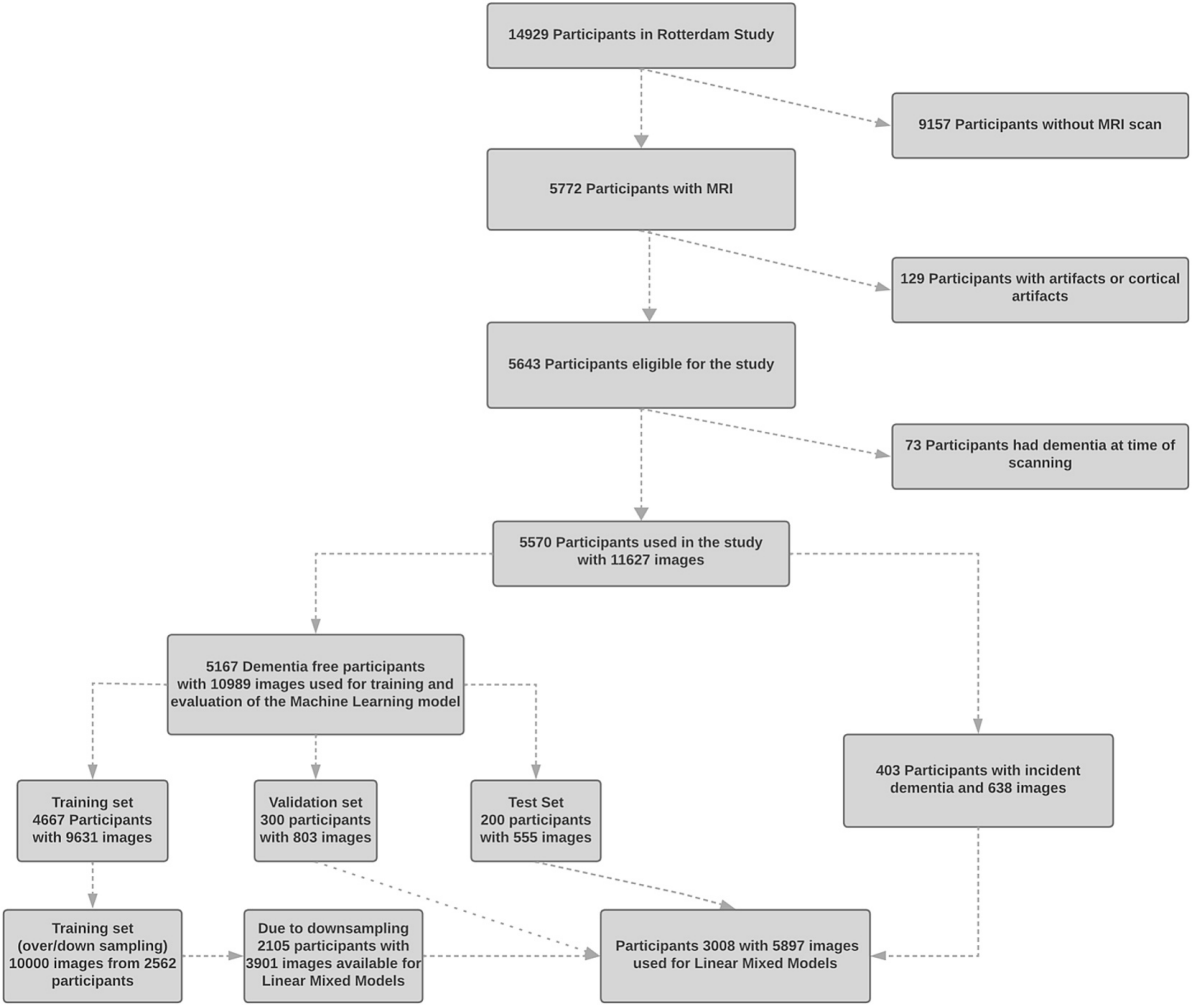
. Flowchart showing the number of participants and the number of MRI brain images per each step.

Our analysis indicates that brain age gap can change meaningfully over time under certain conditions. Specifically, in our longitudinal subgroup, particularly individuals who later developed dementia, we observed a modest yet detectable increase in brain age gap over time (see Fig. A.4). This suggests that brain age gap may indeed be sensitive to accelerated neurodegeneration, even if it remains relatively stable in cognitively healthy individuals. Moreover, we found evidence that higher alcohol consumption is associated with a progressive increase in brain age gap (see Fig. A.5), suggesting that sustained lifestyle exposures may influence brain age gap trajectories.

These findings likely reflect important difference in sample composition and methodology compared to the study by [27]. Our study includes individuals at elevated risk for or progressing toward dementia, whereas their cohorts consisted primarily of healthy participants. Furthermore, the use of a convolutional neural network in our work may offer greater sensitivity to subtle neurodegenerative patterns than more conventional or linear modeling approaches. These distinctions may account for our observation of detectable brain age gap changes over time in specific subgroups.

Second, the lifestyle, education and cardio-metabolic factors explained 21 % of the total variation in brain age gap. Recent studies using neuroimaging-based brain age prediction models have investigated how much variance in the brain age gap can be attributed to demographic, lifestyle, and health-related factors. [28] found that individuals with type 2 diabetes exhibited advanced brain aging, and reported that metabolic risk factors such as BMI and cardiovascular health explained a moderate proportion of brain age gap variance, typically in the range of 10–15 % when combined. More recently, [8] examined a wide range of modifiable risk factors, showing that while individual factors such as cholesterol and physical activity explained only around 2 % of brain age gap variance each, a comprehensive model combining multiple predictors accounted for up to 25 % of the variability. Collectively, these studies emphasize that while individual risk factors often have small effects, their combined impact on brain aging can be substantial and potentially modifiable.

Third, smoking, alcohol, glucose and the use of anti-diabetic medication are associated with a larger brain age gap. In the latter case, the observed increase in brain age gap among individuals using anti-diabetic medication is not probably attributable to the medication itself but rather to the underlying glucose-related conditions that necessitate its use. Moreover, smoking and alcohol consumption are responsible of greater rates of brain atrophy [29,30], a reduction of brain volume and neuronal loss [31]. High blood glucose levels can affect the brain’s functional connectivity, which links brain regions that share functional properties, and brain matter. It can cause the brain to atrophy or shrink [28]. These detrimental effects can be captured by MRI brain images and as a consequence by the concept of brain age gap.

Fourth, the brain age gap is not associated with education, sleeping, cholesterol and blood pressure. This is in line with prior research. Prior studies have suggested that education is an important determinant of cognitive reserve in old age [32], [33]. However, education is not connected with preventing brain atrophy or preventing the shrinkage of the brain [34], [35] and this study is in line with that finding. Furthermore, prior research [36] suggests that sleep duration was not consistently associated with accelerated brain aging as measured by neuroimaging markers such as gray matter volume. Finally, prior work found no significant association between total cholesterol and cognitive decline [37]. Prior work on the association of blood pressure with cognitive decline and dementia reported mixed findings [38].

A limitation of this study is that the lack of a significant association between blood pressure and brain aging observed in this study may be attributable to the method of blood pressure measurement. In the Rotterdam Study, blood pressure was measured at a single time point rather than continuously over a 12 or 24-hour period. This approach may not fully capture the variability in blood pressure or provide an accurate representation of an individual’s overall blood pressure profile, such as nocturnal blood pressure patterns or fluctuations. These limitations could reduce the sensitivity of the analysis in detecting potential associations between blood pressure and brain aging. Additional limitation of the current study is that only gray matter features were used to estimate the brain age gap, potentially overlooking valuable information carried by white matter metrics. White matter microstructural changes are known to reflect age-related processes and have been shown to improve brain age prediction in previous studies [39] Including white matter derived features in future models could enhance prediction accuracy and provide a more comprehensive view of brain aging. Furthermore, the relationship of blood pressure with aging is stronger in studies with a long follow up and with blood pressure measurements in midlife 38. The Rotterdam Study contains participants with high average age and does not reflect to a total exposure to elevated blood pressures throughout a lifespan.

This study investigated how major lifestyle and cardio-metabolic factors contribute to brain age gap. We employed a convolutional neural network trained on healthy participants’ MRI images to calculate brain age gaps in our subsample. Dementia diseased participants appear high values of brain age gap and an increase over time. The factors applied in our study explain 21 % of the total variance in brain age gap, suggesting that unmeasured factors like pollution, diet, sleep quality, physical activity, and genetic factors may play a crucial role. Additionally, our findings indicate that smoking, alcohol consumption, and glucose levels significantly contribute to an increased brain age gap, aligning with previous research linking these factors to brain atrophy and cognitive decline. Conversely, early education, sleep duration, cholesterol, and blood pressure did not show significant associations with brain aging in our study. This may be due to methodological limitations, such as single-time-point blood pressure measurements and a high average age of participants in the Rotterdam Study.

## Data availability

Data from the Rotterdam Study are not publicly available due to informed consent and legal restrictions (e.g., General Data Protection Regulation law in the European Union). However, specific requests for access to the data can be addressed to the Rotterdam Study Management Team that assesses the proposals and adjudicates access—in line with national and international regulations—on a case-by-case basis.

## CRediT authorship contribution statement

**Kostas Stoitsas:** Writing – original draft, Visualization, Software, Investigation, Formal analysis. **Pieter Bakx:** Writing – review & editing, Supervision, Project administration, Conceptualization. **Trudy Voortman:** Writing – review & editing, Resources, Project administration. **Jing Yu:** Writing – review & editing, Software, Resources. **Gennady Roshchupkin:** Writing – review & editing, Supervision, Software, Resources, Conceptualization. **Daniel Bos:** Writing – review & editing, Supervision, Project administration, Methodology, Conceptualization.

## Funding

This work was supported by the A Lifecourse and Individual-based View on Lifestyle to Enhance Health (ALIVE) flagship, funded by the Convergence, the alliance between Erasmus MC University Medical Center Rotterdam, Erasmus University Rotterdam and Delft University of Technology. G.V. Roshchupkin supported by the ZonMw Veni grant (Veni, 1936320).

## Declaration of competing interest

The authors declare that they have no known competing financial interests or personal relationships that could have appeared to influence the work reported in this paper.

## Figures and Tables

**Fig. A.1:**
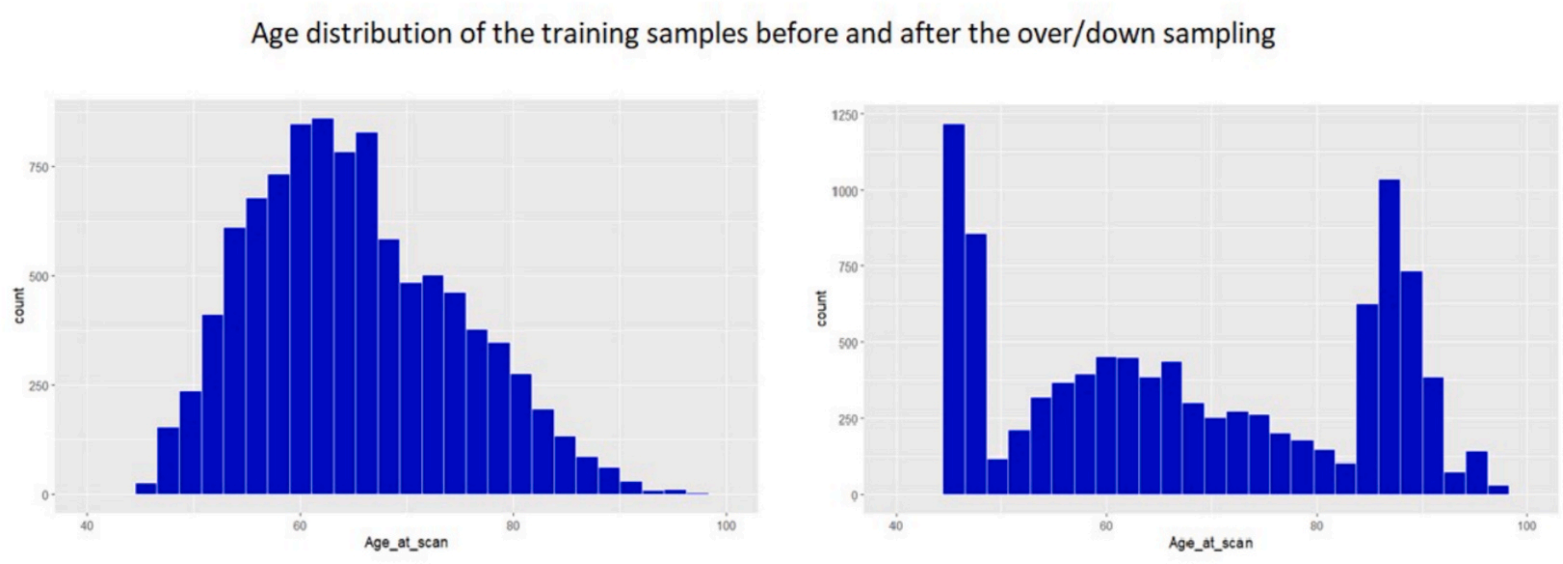
. Age distributions of participants for the training dataset (left) and age distribution after over/down sampling.

**Table:** 

**Model**	**Over/down samplingLearning rateMAE Validation (std)Spearman Correlation ValidationMAE Test (std)Spearman Correlation Test**					
Wang Model	yes	unknown	unknown	unknown	4.45 (3.59)	unknown
Wang Model (32 neurons)	no	0.001	4.33 (5.39)	−0.67	4.83 (5.98)	−0.74
Wang Model (32 neurons)	yes	0.001	4.25 (5.58)	−0.63	4.80 (5.82)	−0.68
Wang Model (32 neurons)	no	0.0005	4.20 (5.41)	−0.58	4.25 (5.86)	−0.63
Wang Model (32 neurons)	yes	0.0005	4.12 (5.21)	−0.55	4.20 (5.65)	−0.59
Wang Model with one extra convolutional layer (32 neurons)	yes	0.0005	4.01 (5.18)	−0.52	4.15 (5.57)	−0.56
Wang Model with one extra convolutional layer (neurons 256)	yes	0.0005	3.99 (5.09)	−0.50	4.06 (5.30)	−0.52
**Wang Model with two extra convolutional layers (neurons 256)**	**yes**	**0.0005**	**3.75 (5.01)**	**−0.42**	**3.88 (5.25)**	**−0.47**

**Table:** 

**Characteristic**	**Regression sample no imputation**	**Regression sample imputed**
Alcohol gr per day (mean, sd)	(9.55, 11.34)	(9.68, 11.58)
Smoking percentage	31.80 %	33.23 %
High education percentage	23.88 %	22.06 %
Systolic blood pressure in mm Hg (mean, sd)	(137.32, 19.53)	(136.96, 19.72)
Diastolic blood pressure in mm Hg (mean, sd)	(81.32, 10.83)	(81.76, 11.06)
Glucose mmol/lt (mean, sd)	(5.65, 1.20)	(5.72, 1.19)
Cholesterol mmol/lt (mean, sd)	(5.66, 1.03)	(5.67, 1.02)
Sleeping hrs per night (mean, sd)	(6.85, 1.23)	(6.88, 1.25)
Sex Percentage Female	53.80 %	53.80 %
Age (mean, sd)	(67.22, 8.82)	(67.22, 8.82)
Observations	5897	5897
Unique individuals	3008	3008

**Fig. A.2. Predicted versus real age for the validation set for the case with lowest MAE. Validation set with 300 subjects and 803 images:**
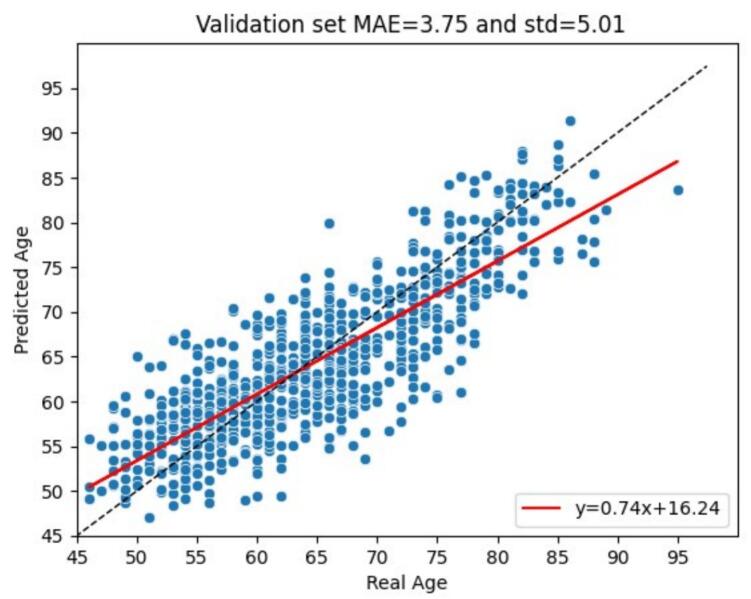


**Fig. A.3:**
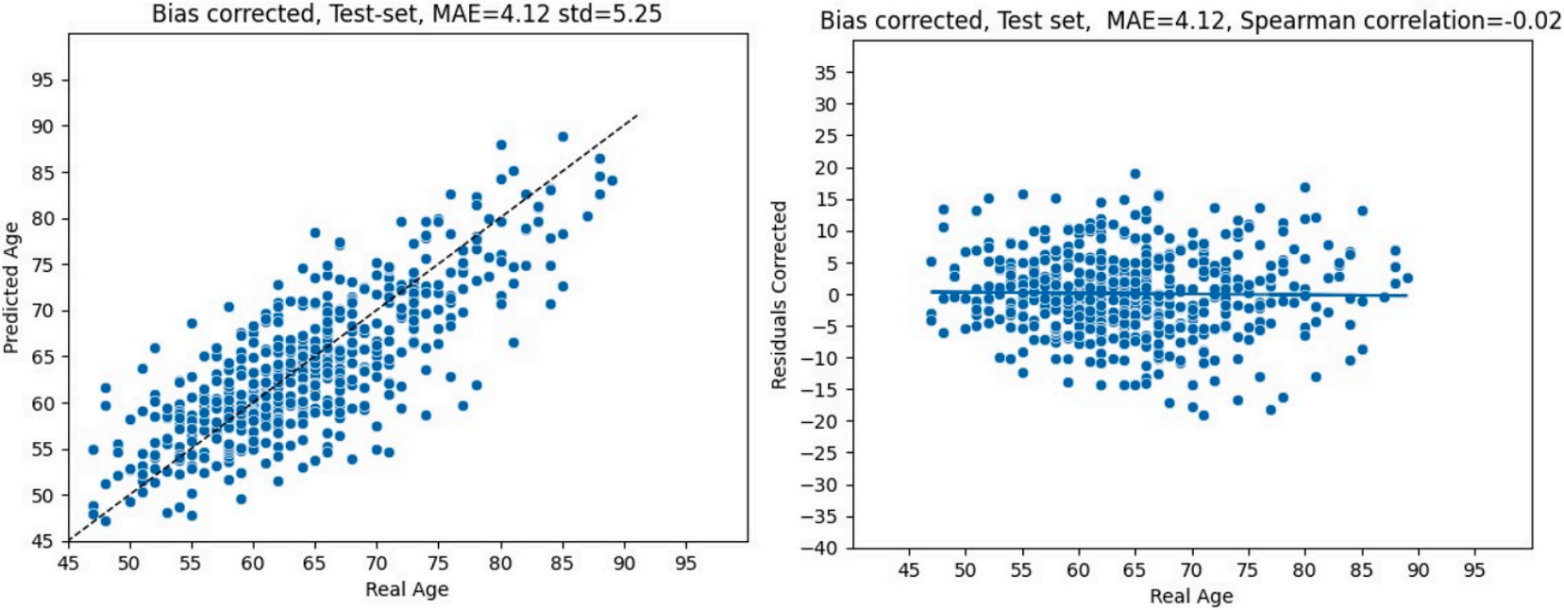
. Predicted age corrected versus real age for the test set (left) and Residuals corrected versus real (chronological) age (right) for the case with lowest MAE on the test set. Test set with 200 subjects and 555 images

**Fig. A.6:**
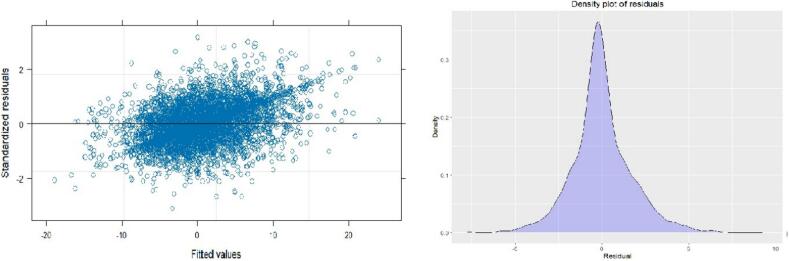
. Residuals over fitted value (left) and density plot of residuals (right) for linear mixed models regression of life habits and education.

**Fig. A.7:**
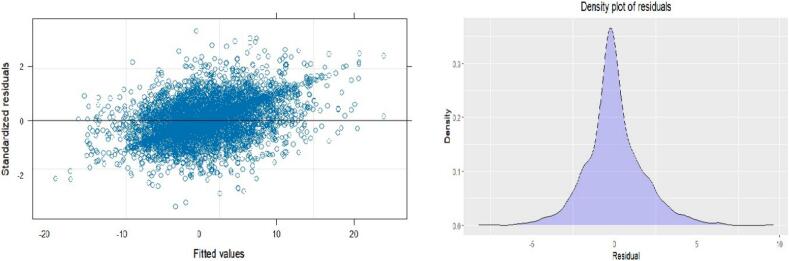
. Residuals over fitted value (left) and density plot of residuals (right) for the linear mixed models regression of cardio-metabolic factors.

**Table:** 

**Characteristic**	**Training**	**Validation**	**Test**
Alcohol gr per day (mean, sd)	(9.27, 10.59)	(9.06, 9.61)	(9.02, 11.11)
Smoking percentage	28.30 %	25.07 %	29.55 %
High education percentage	26.75 %	28.40 %	24.94 %
Systolic blood pressure in mm Hg (mean, sd)	(135.67, 18.75)	(134.74, 19.46)	(134.55, 19.32)
Diastolic blood pressure in mm Hg (mean, sd)	(81.83, 10.70)	(81.93, 10.03)	(81.06, 10.69)
Glucose mmol/lt (mean, sd)	(5.60, 1.17)	(5.68, 1.22)	(5.58, 1.04)
Cholesterol mmol/lt (mean, sd)	(5.66, 1.04)	(5.66, 1.13)	(5.68, 0.98)
Sleeping hrs per night (mean, sd)	(6.88, 1.21)	(6.85, 1.29)	(6.76, 1.25)
Sex Percentage Female	54.28 %	54.55 %	53.67 %
Age	(64.03, 8.04)	(64.76, 8.09)	(64.55, 7.01)

**Fig. A.8:**
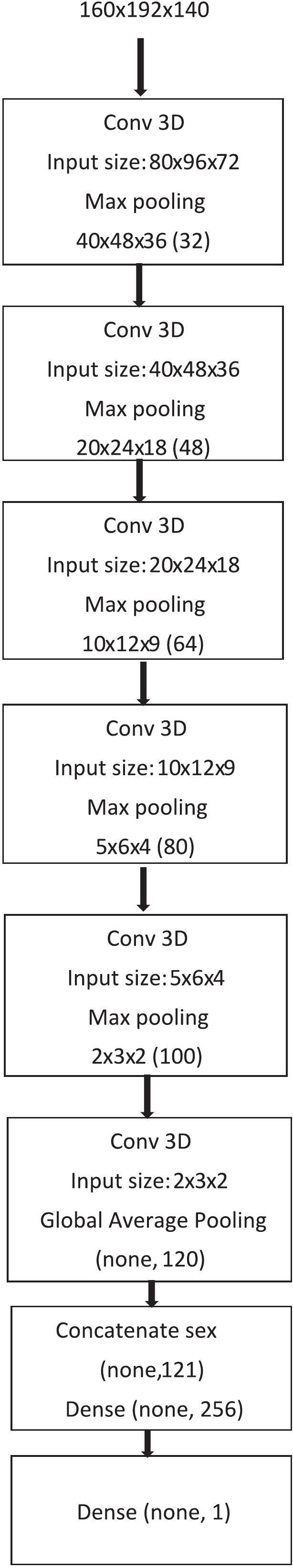
. Structure of CNN with six convolutional layers and 256 neurons at the output layer. *Statistical tests, Appendix A1.* A one-way multivariate analysis of variance (MANOVA) was conducted to examine whether there are differences between full sample (n = 11,627) and regression sample (n = 5,897) across seven independent continuous variables: alcohol gr per day, systolic blood pressure, diastolic blood pressure, glucose, cholesterol, sleeping hours per night, and age were jointly statistically significant.

## Appendix A

.

Table A.1. Optimization experiments to reduce the Mean Absolute Error of the model.

Table A.2. Descriptive statistics for regression subsample with no imputation and with imputation. A one-way multivariate analysis of variance (MANOVA) was conducted to examine whether there are differences between regression (no imputed) and regression (imputed) across seven independent continuous variables: alcohol gr per day, systolic blood pressure, diastolic blood pressure, glucose, cholesterol, sleeping hours per night, and age. The multivariate test was not statistically significant, indicating no overall difference in the multivariate profile between the two datasets: Wilks’ Λ = 0.998, F(7, 11,786) = 1.55, p = 0.140, partial η2 = 0.001. Chi-square tests of independence were conducted to compare smoking status and education level distribution between the imputed and the non-imputed regression dataset. The result was not statistically significant for smoking, χ2(1, N = 11,794) = 2.65, p = 0.103. The effect size, measured by Cramér’s V, was 0.015, indicating a very small association between group membership and smoking status. The difference was not statistically significant for education, χ2(1, N = 11,794) = 3.03, p = 0.082, suggesting no strong evidence of an association between group membership and education level.

Table A.3. General characteristics of participants used in training (no over/down sampling), validation and test sets.Input size image.

The multivariate test was not statistically significant, indicating no overall difference in the multivariate profile between the two datasets: Wilks’ Λ = 0.998, F(7, 17116) = 1.41, p = 0.19, partial *η*^2^ = 0.0006.

Chi-square tests of independence were conducted to compare smoking status, education level, and sex distribution between the full dataset (n = 11,627) and the regression dataset (n = 5897).

A significant difference was observed in smoking status, *X*^2^(1, N = 17,524) = 10.58, p = 0.0011, Craḿer’s V = 0.025, indicating a small effect.

There was no significant difference in education level, *X*^2^(1, N = 8578) = 0.528, p = 0.468, Craḿer’s V = 0.008.

Sex distribution was also not significantly different, *X*^2^(1, N = 8578) = 0.216, p = 0.642, Craḿer’s V = 0.005.
